# An End-to-End Deep Learning Approach for State Recognition of Multifunction Radars

**DOI:** 10.3390/s22134980

**Published:** 2022-07-01

**Authors:** Xinsong Xu, Daping Bi, Jifei Pan

**Affiliations:** College of Electronic Engineering, National University of Defense Technology, Hefei 230037, China; dapeei@163.com (D.B.); panjifei17@nudt.edu.cn (J.P.)

**Keywords:** multifunction radar, radar signal recognition, radar state recognition, recurrent neural network

## Abstract

With the widespread use of multifunction radars (MFRs), it is hard for the traditional radar signal recognition technology to meet the needs of current electronic intelligence systems. For signal recognition of an MFR, it is necessary to identify not only the type or individual of the emitter but also its current state. Existing methods identify MFR states through hierarchical modeling, but most of them rely heavily on prior information. In the paper, we focus on the MFR state recognition with actual intercepted MFR signals and develop it by introducing recurrent neural networks (RNNs) of deep learning into the modeling of MFR signals. According to the layered MFR signal architecture, we propose a novel end-to-end state recognition approach with two RNNs’ connections. This approach makes full use of RNNs’ ability to directly tackle corrupted data and automatically learn the features from input data. So, it is practical and less dependent on prior information. In addition, the hierarchical modeling method applied to the end-to-end network effectively restricts the scale of the end-to-end model so that the model can be trained with a small amount of data. Simulation results on a real MFR show the excellent recognition performance of our end-to-end approach with little prior information.

## 1. Introduction

Radar signal recognition, one of the key technologies of modern electronic intelligence systems, plays an important role in modem electronic warfare. Traditional radar signal recognition mainly uses statistical pattern recognition approaches to identify the type or individual of the emitter to which the target signal belongs and thus evaluate the potential threats, namely radar emitter classification (REC) and specific emitter identification (SEI) [1,2,3]. With the development of technology, multifunction radars (MFRs) have been widely deployed. These radars are complex sensors that can transform their functional states and waveforms actively and adaptively to perform different tasks [4]. They pose a significant challenge to traditional radar signal reconnaissance systems. For the MFR signal processing, it is necessary to find more effective ways to cope with the exploding parameter space of MRF signals and further identify their current states [5].

Research on MFR state recognition can be traced back to the syntactic modeling of MFRs by Visnevski et al. in [6,7]. In their study, MFRs are regarded as stochastic discrete event systems and characterized by stochastic context-free grammars. To deal with the complex signal structures of MFRs, they propose a hierarchical modeling approach in which the MFR signal is described layered as radar phrases, radar words, and radar pulses. Their research opens the way for studies on MFR state recognition, and most of the subsequent research is based on their framework. According to the idea of hierarchical modeling, MFR state recognition can be completed in two steps. The first step is to identify radar words from input pulse sequences, and the second step is to identify MFR states from the identified word sequences. At present, radar word recognition and state recognition are independent. The radar word recognition methods mainly include the modeling method [8], event-driven method [7], and matched filtering method [9]. All these methods require the accurate radar word templates, which rely heavily on prior knowledge. The early state recognition methods are based on the syntactic modeling of MFRs, which is to model the entire radar system [5]. This requires detailed intelligence information about the scheduling and generation of radar signals, which is not easy in practice. In references [10,11], MFR signal sequences are viewed as stochastic discrete dynamic processes, and they use target modeling instead of radar modeling to infer radar states. The models used are observable operator models (OOMs) [12] and predictive state representation (PSR) models [13]. The target modeling is for the received signals from the signal receiver’s point of view. The advantage is that it does not require complete MFR system information and is more practical.

In the field of radar countermeasures, MFR state recognition has attracted more and more attention, and some breakthroughs have been made. However, traditional methods rely heavily on prior information. In the actual state recognition, only a certain amount of corrupted radar signal data can be used, and the parameter information of MFRs is extremely well protected. Therefore, how to extract useful features from the corrupted MFR signals and identify the MFR states in the case of limited prior information remain significant issues to solve. With the arrival of the big data era and the rapid development of artificial intelligence, machine learning and deep learning are widely used. Their ability to automatically learn the effective features from raw data has achieved great success in the computer domain and other fields. Deep learning is also increasingly used in radar signal processing [14,15,16]. For MFR recognition, more and more papers use recurrent neural networks (RNNs) [17] to process the sequence data of MFRs [18,19,20]. Inspired by them, we introduced RNN to identify MFR states from extracted radar word sequences in our paper [21]. Compared with OOM or PSR, the advantage of RNNs is that they can process raw data directly and learn the effective features from it without other designs. Moreover, RNNs are nonlinear models that can better tackle corrupted radar signal data.

Based on the work of our paper [21], we add the radar word recognition to the network of MFR state recognition in this paper and propose a novel end-to-end recognition model for MFR state recognition. The whole network is the concatenation of two RNNs: the first RNN extracts the representation vectors of radar words from the input pulse sequences, which can be regarded as a feature extractor; the second RNN acts as a state recognizer to recognize the MFR states from the extracted representation vectors. The two RNNs form a complete end-to-end recognition model through joint training and optimization. We train the end-to-end network step by step. We first train the feature extraction network, fix the parameters of this network, and then add the state recognition network for the complete end-to-end model training. Using two RNNs comes from the idea of hierarchical modeling, which can enable us to keep the overall level of complexity of MFR models manageable [7]. Our training can also effectively control the convergence direction of model training as it adds the constraint of radar words in the middle of the network. The design dramatically reduces the amount of data required for training.

To the best of our knowledge, we are the first to propose an end-to-end model for identifying MFR states from radar pulse sequences. The end-to-end approach can increase recognition accuracy through joint training and optimization. Moreover, it is easy to implement and greatly improves the practicability of the MFR state recognition. The MFR state recognition results on the MFR called Mercury show that our end-to-end recognition method achieves a high recognition accuracy with less prior information.

The rest of the paper consists of five parts. Section 2 introduces the state recognition process of an MFR and proposes the end-to-end recognition approach based on RNNs. In Section 3, the details of the end-to-end recognition approach are given. Section 4 verifies the performance of the approach via simulations. The conclusions are given in Section 5.

## 2. MFR State Recognition

### 2.1. Layered Radar Signal Architecture

**Figure 1:**
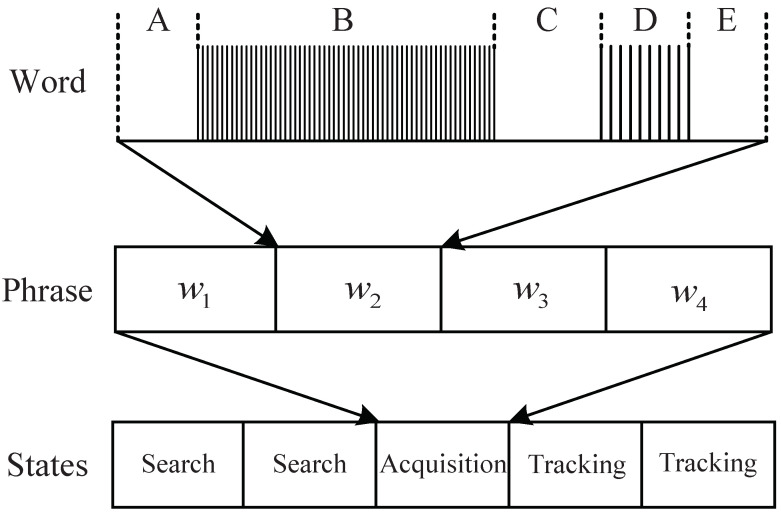
A common layered MFR signal structure.

MFRs employ multiple functional states and sophisticated signal waveforms to detect targets. In the study of syntactic modeling, Visnevski et al. developed a layered signal model following the commonly used MFR signal structure [5]. MFR signals are described hierarchically by pulses, words, and phrases. Radar pulses are the actual physical pulses that can be expressed by parameters such as carrier frequency (CF), arrival time (TOA), pulse repetition interval (PRI), pulse width (PW), etc. Radar words represent fixed arrangements of a finite number of pulses that are optimized for extracting a particular target information. Radar phrases are concatenations of a finite number of radar words, and each phrase corresponds to a radar state such as search or tracking. Figure 1 shows an example of the signal model [7]. It is a real anti-aircraft MFR and has a three-layer signal structure that is common to many MFRs. In the figure, we can see that a radar word of the MFR comprises five sections (A–E). Sections A–E are the dead time of fixed duration. Section B is a fixed PRI pulse-doppler sequence, and section D is a synchronization burst. Different radar words are different in section B. Radar phrases consist of four radar words, and each is associated with a single state.

### 2.2. MFR State Recognition Process

The traditional radar signal reconnaissance process is as follows [5]: First, radar pulses are detected by the radar antenna, and their parameters are measured in the radar receiver. Then, the radar deinterleaver processes the parameters and groups the pulses according to their possible emitters. At last, the analyzer performs REC or SEI for the deinterleaved pulses and thus assesses the threat.

**Figure 2:**
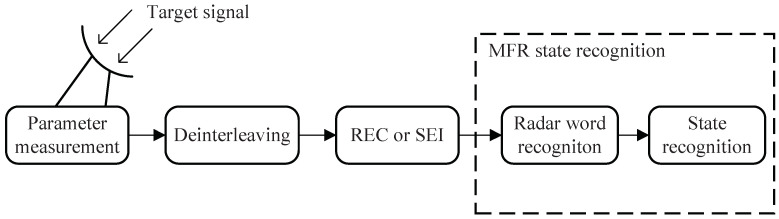
MFR state recognition process.

As an MFR can conduct multiple tasks simultaneously and emit waveforms with sophisticated signal structures, the traditional radar signal recognition is inadequate. In order to estimate the threat of an MFR, it is essential to go further to identify its current states. The idea of hierarchical modeling [7] offers a good scheme for MFR state recognition. According to the layered MFR signal structure, MFR state recognition can be carried up in two steps. The first step is to extract radar words from pulse sequences, and the second step is to identify radar states from the extracted word sequences. They can be viewed as radar word recognition and state recognition. The whole process of MFR state recognition is illustrated in Figure 2.

### 2.3. MFR State Recognition Based on the End-to-End Deep Learning Network

In the existing papers, radar word recognition and state recognition are independent, and their recognition methods rely heavily on prior information. For example, radar word recognition requires radar word templates, and state recognition needs information such as the length and the list of radar phrases in each state. In practice, however, this information is extremely well protected. There is only a certain amount of radar signal data intercepted by the radar reconnaissance system that can be used. Hence, we need to focus on the actual intercepted MFR signal and propose a more practical approach for the MFR state recognition.

Deep learning [22] is widely used in data processing and has achieved great success in speech recognition, computer vision, natural language processing, etc. As a representation learning method, deep learning can automatically and deeply learn the useful features of raw data. This allows it to gradually supersede the feature engineering [17] and shallow neural network. Inspired by its success, we introduced deep learning into the actual radar signal processing and used RNNs in our MFR state recognition [21]. RNNs are neural networks with the hidden states that can capture the historical information of previous time steps. In deep learning, convolutional neural networks (CNNs) are designed for spatial information processing, while RNNs are for sequence data processing. The gated recurrent units (GRU) [23] is one of the most commonly used RNNs. It is a variant of simple RNNs which is designed to tackle the vanishing gradient problem. Now, GRUs are also widely used in the field of radar [24,25].

We have applied a GRU to the state recognition of radar word sequences in our paper [21], achieving pretty good results. This time, we also apply the GRU to the radar word recognition and offer a complete end-to-end recognition network. Compared with the previous state recognition methods, the advantages of GRU-based state recognition methods are the following [21]: (1) GRUs can process raw data directly without the need of other designs, and its ability to learn the features from the input data reduces the dependence on prior information; (2) GRUs can capture long dependencies in sequences and are more suitable for modeling long sequences such as radar pulses; (3) GRUs are nonlinear models that can better tackle the corrupted radar signal as well as the sequence alignment problems caused by missing data.

**Figure 3:**
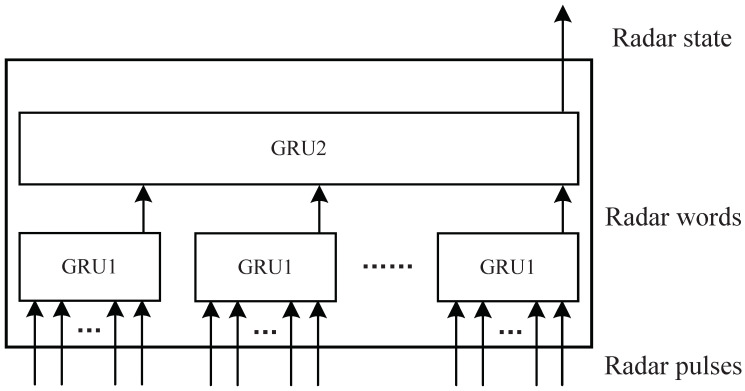
End-to-end recognition network.

For complete MFR state recognition, another advantage of RNNs is that two neural networks can be connected directly and trained together. In the paper, we identify MFR states with two GRUs and connect them to form an end-to-end recognition network model. The end-to-end network can significantly speed up data processing and make online processing possible. In our end-to-end recognition approach, the input is the radar pulse sequences represented by PRI parameters. The output is the radar states such as search, acquisition, tracking, etc. We adopt the idea of hierarchical modeling and use the radar words in the middle of the two GRUs. That is, we use a GRU to extract the representation vectors of radar words from the input pulse sequences and the other GRU to identify the MFR states from the extracted radar word sequences. The end-to-end network is shown in Figure 3. In a broad sense, radar word recognition can be regarded as the feature extraction of input pulse sequences. The entire process of MFR state recognition is feature extraction and state recognition. The advantage of hierarchical modeling is that the complexity of the MFR signal model can be manageable [7]. Similarly, the hierarchical network that introduces constraints of radar words in the middle of the network can effectively control the convergence direction of the model and greatly reduce the amount of data required for training. This is very important for actual MFR state recognition, as it is difficult to obtain enough data. This is the reason why we do not directly use one GRU to recognize the radar state from the radar pulse sequences.

## 3. End-to-End Recognition Approach

### 3.1. Representation of Radar Pulse Sequences

Radar pulse sequences are usually represented by the parameter set of TOA or PRI. PRI cannot be measured directly but also needs to be obtained through TOA. The TOAs of pulses are quantification by a digital receiver, which uses the observer clock denoted as $T_{obs}$ to control the process [8]. Assume that the relative TOA of received pulse is $t_{i}$; then, the theoretical pulse quantization time is

(1) $$T_{i}=n_{i}\cdot T_{obs}=\left\lfloor \frac{t_{i}}{T_{obs}}\right\rfloor \cdot T_{obs}$$

where $T_{i}$ and $n_{i}$ are associated quantization time and index. In practice, the quantization model needs to include a uniformly distributed random phase $\varphi \in \left[0,T_{obs}\right)$ to accommodate for the asynchronous nature of the radar and receiver [8]. So, the practice pulse quantization model is

(2) $$n_{i}^{\prime }\left(\varphi \right)=\left\lfloor \frac{t_{i}+\varphi }{T_{obs}}\right\rfloor =\left\{\begin{array}{cc} n_{i}, & \;\mathrm{with}\;1-p_{i}\\ n_{i}+1, & \;\mathrm{with}\;p_{i} \end{array}\right.$$

where $p_{i}$ is the pulse splitting probability

(3) $$p_{i}=\frac{t_{i}}{T_{obs}}-\left\lfloor \frac{t_{i}}{T_{obs}}\right\rfloor$$

In addition to the quantization error, there are lots of spurious and missing pulses that need to be considered. In the previous radar word recognition methods, radar word templates, the ideal TOA set of the pulse sequences is the important prior information. However, it is unavailable in practical application. They need to be constructed from the corrupted data. Deep learning networks process the raw data directly; thus, it can bypass the construction process of radar word templates. In this paper, we use a GRU to learn the representation vectors of radar words from received radar pulse sequences. It is more direct and efficient.

Here, we use the quantized PRI to represent the input radar pulse sequence *X*

(4) $$X=\left\{x_{1},x_{2},\cdots ,x_{m}\right\},\;x_{i}=n_{i+1}^{\prime }-n_{i}^{\prime }$$

The sequence includes spurious and missing pulses. In order to facilitate the processing of GRU, we map each parameter to a one-hot vector before input.

### 3.2. Radar Word Recognition

**Figure 4:**
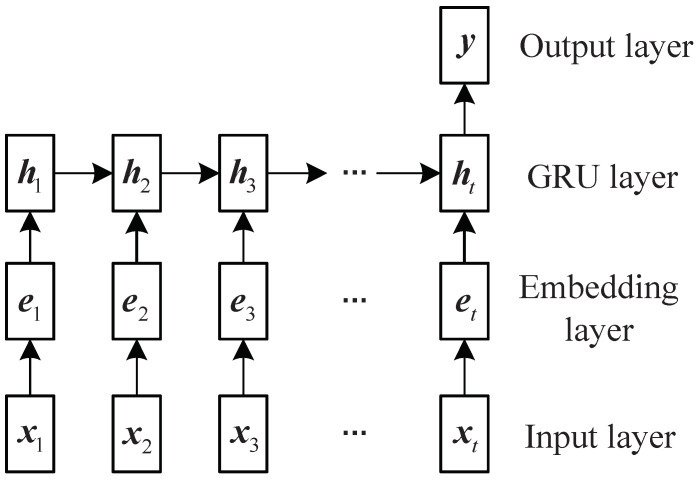
The network architecture of radar word recognition.

We use the GRU to identify radar words. Figure 4 illustrates the network structure. The input is a set of PRI parameters represented by one-hot vectors. In the embedding layer, they are converted into low-dimensional features as follows,

(5) $$e_{t}=W_{xe}x_{t}$$

where $x_{t}\in R^{L\times 1}$ is the input one-hot vector; $W_{xe}\in R^{l\times L}$ is the embedding matrix that is learned by training; $e_{t}\in R^{l\times 1}$ is the embedded vector. It is a dimensionality reduction method commonly used in deep learning [17].

The network extracts the features of sequences in the GRU layer. The GRU can automatically filter and extract the useful information of each time step and pass it to the next step through the hidden state $h_{t}$. In the end, the hidden state captures the whole information of the entire sequence. Detailed calculation procedures of $h_{t}$ are as follows [23],

(6) $$\begin{array}{cc} r_{t} & =\sigma \left(W_{er}e_{t}+W_{hr}h_{t-1}+b_{r}\right) \end{array}$$

(7) $$\begin{array}{cc} z_{t} & =\sigma \left(W_{ez}e_{t}+W_{hz}h_{t-1}+b_{z}\right) \end{array}$$

(8) $$\begin{array}{cc} {\tilde{h}}_{t} & =\tanh\left(W_{eh}e_{t}+W_{hh}\left(r_{t}⊙h_{t-1}\right)+b_{h}\right) \end{array}$$

(9) $$\begin{array}{cc} h_{t} & =z_{t}⊙h_{t-1}+\left(1-z_{t}\right)⊙{\tilde{h}}_{t} \end{array}$$

where $r_{t}$, $z_{t}$, ${\tilde{h}}_{t}$, and $h_{t}$ denote the reset gate, update gate, candidate hidden state, and hidden state; σ is logistic sigmoid function, and the symbol ⊙ indicates element-wise multiplication between tensors; $W_{er}$, $W_{hr}$, $b_{r}$, $W_{ez}$, $W_{hz}$, $b_{z}$, $W_{eh}$, $W_{hh}$, and $b_{h}$ are weight parameters. Compared with a simple RNN, the GRU uses the above learnable gates to control when a hidden state should be updated and when it should be reset.

The output layer is a fully connected layer in which the extracted features are mapped into a fixed-dimensional vector. This is the extracted radar word representation vector used as input for the following state recognition. In order to facilitate training and verification, we perform the softmax operation on the representation vector in the output layer. Softmax is used as the last activation function of a neural network to normalize the output to a probability distribution over the output categories. It transforms the outputs such that they become non-negative and sum to 1 [17]. The calculation formula of the outputs is

(10) $$y=\mathrm{softmax}\left(W_{hy}h_{t}+b_{y}\right)$$

where $W_{hy}$ and $b_{y}$ are weight parameters to be trained.

### 3.3. State Recognition

**Figure 5:**
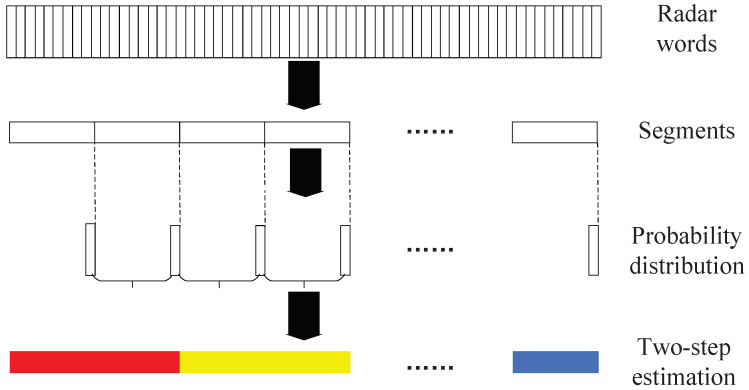
A flowchart of the state recognition.

In our paper [21], we described the detailed state recognition process from radar word sequences to radar phrase sequences. In the end-to-end recognition network model, it just needs to change the input of the state recognition network and use the representation vectors of radar words to connect the radar word recognition network and the state recognition network. The whole process of state recognition is shown in Figure 5 [21].

Firstly, we divided the extracted radar word sequence into segments, each of which corresponds to a radar state to be identified. We set the segment length as a hyperparameter and verify the recognition performance of different lengths in the experiments.

Secondly, the segments are input into the GRU for feature extraction and the probability estimation of the output category. The GRU is a nonlinear model that can effectively tackle the alignment problem caused by the missing radar words. The output is probability distribution $p\left(z_{n}=s_{i}|y_{1:T}^{n}\right)$, where $y_{1:T}^{n}$ is the input radar word segment and $z_{n}$ is the output state variable.

Finally, each state is identified by our two-step estimation method. This method comes from the idea of accumulating the predictive states [11], and we simplify its process by using the probability distribution of the previous and current two segments to estimate the state of the current segment. This is based on the knowledge that the MFR state remains unchanged for a short time. The two-step estimation method improves the recognition accuracy, but it also brings a certain recognition delay when the state switches. The final state is identified as follows

(11) $$\begin{array}{c} p\left(z_{n}=s_{i}\right)=\frac{p\left(z_{n}=s_{i}|y_{1:T}^{n}\right)p\left(z_{n-1}=s_{i}|y_{1:T}^{n-1}\right)}{\sum_{j=1}^{r}p\left(z_{n}=s_{j}|y_{1:T}^{n}\right)p\left(z_{n-1}=s_{j}|y_{1:T}^{n-1}\right)} \end{array}$$

(12) $$\begin{array}{c} {\hat{z}}_{MAP}=\arg\left(\max_{1\leq i\leq r}p\left(z_{n}=s_{i}\right)\right) \end{array}$$

### 3.4. Training of the End-to-End Model

The values of model parameters are determined through supervised training. Since our end-to-end model consists of the radar word recognition network and state recognition network, we train the end-to-end network step by step. We first train the radar word recognition network, then fix the parameters of this trained network, and finally join the state recognition network for the complete end-to-end training. Correspondingly, the training set consists of radar pulse sequences, radar word labels, and radar state labels. We use radar pulse sequences and corresponding radar word labels to train the radar word recognition network and use radar pulse sequences and corresponding state labels to train the end-to-end network. This training method allows us to train the model with a small amount of data as it keeps the level of complexity of the model manageable.

Before training, a loss function [17] is required to measure the error of the model. The training process of the model is the process of minimizing the loss function. We use the cross-entropy loss function often used in classification problems in our model training. In the end, we train the radar word recognition network to minimize the negative log-likelihood

(13) $$\min_{\theta }\frac{1}{N}\sum_{n=1}^{N}-\log p_{\theta }\left(y_{n}|x_{n}\right)$$

where $\left(x_{n},y_{n}\right)$ is a (radar pulse sequences, radar word) pair in the training set; *N* and θ denote the number of the pairs and the parameter set. We train the end-to-end network to minimize the negative log-likelihood

(14) $$\min_{\varphi }\frac{1}{M}\sum_{m=1}^{M}-\log p_{\varphi }\left(z_{m}|x_{m}\right)$$

where $\left(x_{m},z_{m}\right)$ is a (radar pulse sequences, radar states) pair in the training set; *M* and φ denote the number of the pairs and the parameter set. It is worth noting that there are *T* words in the input $x_{m}$, where *T* is the length of segments.

In addition to the loss function, the optimization algorithm is another key to model training. We employ gradient-based optimization algorithms and backpropagation algorithms [17], which are commonly used in deep learning to train our model.

## 4. Simulations

In this section, a series of simulation experiments are carried out to test the effectiveness of our approach. All the simulations are based on the Mercury radar given in [5,8,11,26], which is the most commonly used MFR in the research of MFR signal processing.

### 4.1. Mercury Radar

**Figure 6:**
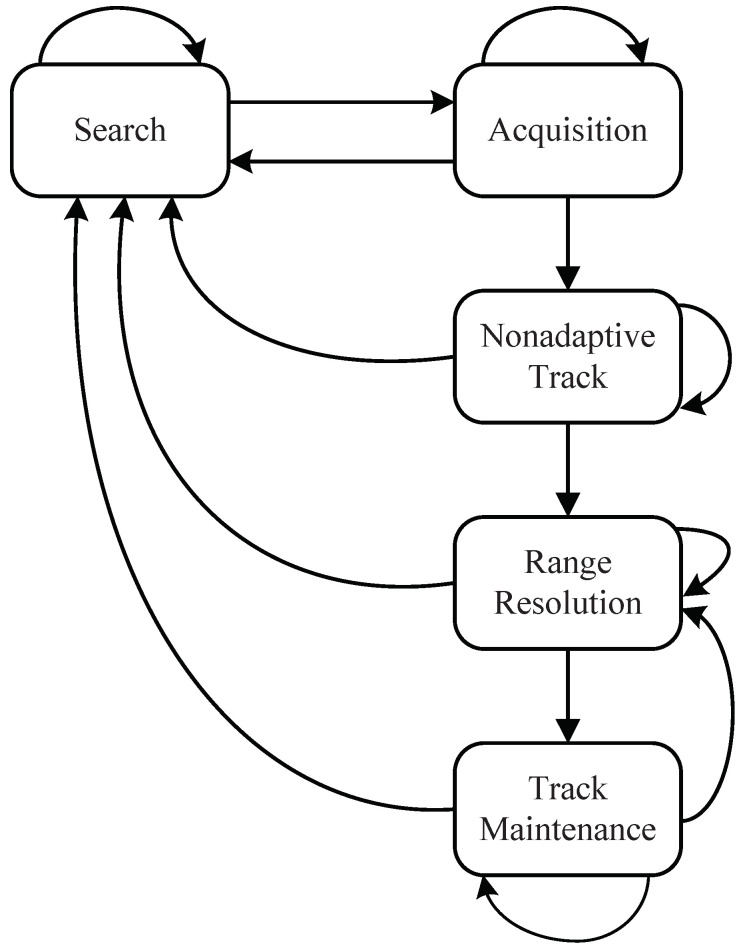
State transition of the Mercury radar.

The Mercury radar is a ground-based MFR whose primary mission is to provide anti-aircraft defense. Visnevski et al. released the functional specification of the radar in their research on MFR syntactic modeling [7]. We have introduced the word structure of the Mercury radar in Section 2.1 and Figure 1. The Mercury radar can emit nine different radar words, and the signal length of each word is the same: 7.14 ms. The difference between each word is the PRI in section B, which is less than 100 μs. The Mercury radar is an MFR capable of performing different tasks using radar phrases. Each phrase is a sequence of four consecutive radar words allocated to one state. The specification discloses the five functional states of the Mercury radar—search, acquisition, nonadaptive track (NAT), range resolution (RR), and track maintenance (TM). The transitions between states are shown in Figure 6 [5], and the list of all the phrases of the Mercury radar according to each functional state is illustrated in Table 1 [5]. The symbols $w_{1}$, $w_{2}$, *…*, $w_{9}$ denote the nine words. In our simulations, the training set and test set are generated according to the table and the nine radar words, but it is worth noting that the information is not prior knowledge.

### 4.2. Simulation Settings

In the simulations, we generate a radar pulse train consisting of 500 radar phrases for each state of the Mercury radar. They will be used as the training set for model training. The radar phrases for each state are selected randomly from the list in Table 1. A certain number of radar words in each sequence are lost to simulate real received radar signal data. For the radar words in the sequence, we each generate a radar pulse sequence according to the quantization model described in Section 3.1. The spurious pulses and missing pulses are considered in the pulse sequences. We conduct a series of experiments to test the radar word recognition performance with different levels of corrupted training data.

The training set consists of radar states, radar word sequences, and radar pulse sequences. We first use the radar words in all phrase sequences and the corresponding radar pulse sequences to train the radar word recognition network. Then, we use the radar states and the corresponding radar pulse sequences to train the end-to-end network. To train the end-to-end network, we need to divide the entire sequence into segments of the fixed radar word length. We specially make five datasets of different lengths to verify the recognition performance.

The test set is also a pulse train of 500 radar phrases, but it includes all the five states of search, acquisition, NAT, RR, and TM. The radar phrases in each state are randomly selected according to Table 1. For the test set, a certain number of radar words are also randomly lost, and the radar pulse sequence corresponding to each radar word is generated in the same way as the training set.

All the training and testing are on the PyTorch platform. The hyperparameters of the network are tuned during training. After tuning the hyperparameters, we find that the model performance is not sensitive to the layer size of the GRU hidden layer, and we employ two hidden layers with 128 units each for the first GRU. The output size of the GRU is nine, as there are nine radar words. For the second GRU, it is also two hidden layers with 128 hidden units each. The input and output units of the fully connected layer are 128 and 5 because it is connected to the hidden layer of the second GRU and outputs five MFR states. The optimization algorithm used in training is Adam.

### 4.3. Results

#### 4.3.1. Performance of the Radar Word Recognition

We first use the radar pulse sequences and the radar words to train and verify the network performance of the radar word recognition. To study the influence of corrupted data, the data with different spur rates and missed rates are used for training and verifying. Table 2 shows the results. From the table, we can see that the recognition accuracy is more than 90% even when the percentage of missed pulses is 30%, and the spur rate is 30,000 pulses/s. When the data are seriously corrupted, the recognition accuracy declines rapidly. This is because the number of spurious pulses far exceeds the number of actual radar pulses, and the true PRIs are severely distorted. It is worth noting that these are the results without the prior information of radar word templates. Compared with the methods with templates [7,9], the recognition accuracy is lower, but it is enough to meet the performance requirements of the subsequent state recognition and is more suitable in the actual application.

#### 4.3.2. Performance of the End-to-End Network

**Figure 7:**
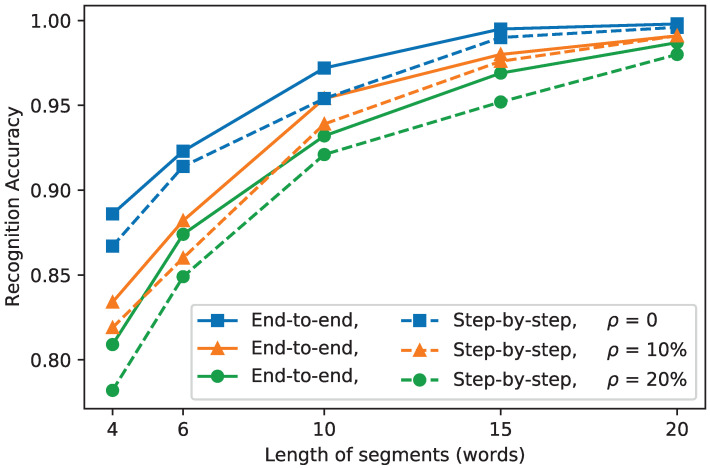
The accuracy of the end-to-end recognition and step-by-step recognition with different segment lengths and radar word loss.

In this section, we further carry out experiments to test the performance of the end-to-end network. Here, we set the percentage of missed pulses as 30% and the spur rate as 30,000 pulses/s in each pulse sequence. In this case, the recognition accuracy of the radar word network is about 90% according to the results above. For the end-to-end network, we verify its performance with different segment lengths and different percentages of missed words. The segment length ranges from four radar words to 20 radar words, and the percentages of missed words ρ are 0%, 10%, and 20%. As a comparison, we also verify the performance of the step-by-step model in which the radar word recognition and state recognition are independent. All the results are illustrated in Figure 7, where each line shows how the recognition accuracy varies with the increasing segment length at the same percentage of missed words. The solid lines are for the end-to-end recognition, while the dashed lines are for the step-by-step recognition. From the figure, we can see that when the length of segments increases and the percentage of missed words decreases, the accuracy of the model increases. This is because the more correct data the model uses, the better the recognition accuracy. The accuracy of the end-to-end recognition is obviously higher than that of the step-by-step recognition with the same segment length and percentage of missed words. We can also see that the alignment problem caused by the loss of radar words has no obvious effect on the performance of our GRU-based network model. In general, our GRU-based end-to-end network can directly process the corrupted MFR signal data and effectively increase the recognition accuracy.

#### 4.3.3. Results of the Complete MFR State Recognition

**Figure 8:**
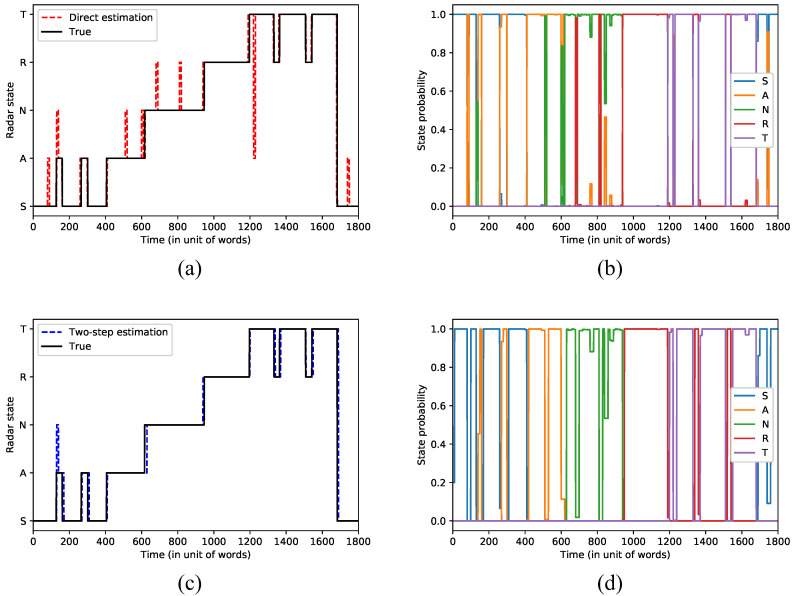
Recognition results of radar states based on the end-to-end network. (**a**) Radar state recognition by direct estimation, (**b**) Probability distribution of radar states by direct estimation, (**c**) Radar state recognition by two-step estimation, (**d**) Probability distribution of radar states by two-step estimation.

Finally, we use the radar pulse sequences and the radar states in the test set to test our method. The test sequence includes the whole five functional states. According to the above performance results of the radar recognition and the end-to-end recognition, we set the main network parameters in the MFR state recognition test as follows: the percentage of missed pulses is 30%; the spur rate is 30,000 pulses/s; the percentage of missed words is 20%; the segment length is 10 radar words. We perform two tests: one is to estimate the radar states directly by the end-to-end network, and the other is to estimate by adding the two-step estimation method to the end-to-end network. Figure 8 shows the results of the two tests, where Figure 8a,b is used for the direct estimation and Figure 8c,d is used for the two-step estimation. The vertical axes of Figure 8a,c display the five states: search (S), acquisition (A), nonadaptive track (N), range resolution (R), and track maintenance (T), and the horizontal axes display the input signal sequence in the unit of words. The dashed lines in Figure 8a,c show the states estimated by the algorithms, and the solid lines show the true states. Figure 8b,d show the probability distribution estimation over states, where each line denotes one state.

As shown in Figure 8a, we can see some outliers in the state recognition results. They are the misidentification results when only one segment is used. The error probability is in line with the performance results of the end-to-end model with the same parameters. Figure 8c shows the state recognition results by the two-step estimation method where the recognition errors disappear. This is because the method employs the information of two segments (20 radar words) for recognition. However, the method also leads to a certain delay in recognition results when the state changes. This can be seen from the result in Figure 8c that the estimated states are slightly behind the true states. In general, our end-to-end recognition approach achieves great recognition accuracy with less prior information.

## 5. Conclusions

In this paper, we introduce RNNs of deep learning into MFR state recognition. We describe the MFR state recognition process and propose an end-to-end recognition approach to identify MFR states from actual intercepted MFR pulse sequences. Our approach takes advantage of GRU to automatically learn the features of sequence data and process corrupted data, which greatly improves the practicability of the MFR state recognition. Moreover, the hierarchical modeling method that we use in the network effectively restricts the scale of the end-to-end model so that we can train the model with a small amount of data. In the experiments, we show the excellent recognition performance of our end-to-end approach with little prior information.

This method has only been tested on the publicly available Mercury radar data. In the future, we will apply our model to more MFRs. For the complete online recognition of MFR states, we also need to make a breakthrough in the online deinterleaving of radar pulse sequences.

## Figures and Tables

**Table 1:** List of all Mercury radar phrase combinations according to the functional states of the radar.

States	Phrases	States	Phrases
Search	$w_{1}w_{2}w_{4}w_{5}$	Track Maintenance	$w_{1}w_{7}w_{7}w_{7}$
	$w_{2}w_{4}w_{5}w_{1}$		$w_{2}w_{7}w_{7}w_{7}$
	$w_{4}w_{5}w_{1}w_{2}$		$w_{3}w_{7}w_{7}w_{7}$
	$w_{5}w_{1}w_{2}w_{4}$		$w_{4}w_{7}w_{7}w_{7}$
	$w_{1}w_{3}w_{5}w_{1}$		$w_{5}w_{7}w_{7}w_{7}$
	$w_{3}w_{5}w_{1}w_{3}$		$w_{6}w_{7}w_{7}w_{7}$
	$w_{5}w_{1}w_{3}w_{5}$		$w_{1}w_{8}w_{8}w_{8}$
Acquisition	$w_{1}w_{1}w_{1}w_{1}$		$w_{2}w_{8}w_{8}w_{8}$
	$w_{2}w_{2}w_{2}w_{2}$		$w_{3}w_{8}w_{8}w_{8}$
	$w_{3}w_{3}w_{3}w_{3}$		$w_{4}w_{8}w_{8}w_{8}$
	$w_{4}w_{4}w_{4}w_{4}$		$w_{5}w_{8}w_{8}w_{8}$
	$w_{5}w_{5}w_{5}w_{5}$		$w_{6}w_{8}w_{8}w_{8}$
Nonadaptive Track or Track Maintenance	$w_{1}w_{6}w_{6}w_{6}$		$w_{1}w_{9}w_{9}w_{9}$
	$w_{2}w_{6}w_{6}w_{6}$		$w_{2}w_{9}w_{9}w_{9}$
	$w_{3}w_{6}w_{6}w_{6}$		$w_{3}w_{9}w_{9}w_{9}$
	$w_{4}w_{6}w_{6}w_{6}$		$w_{4}w_{9}w_{9}w_{9}$
	$w_{5}w_{6}w_{6}w_{6}$		$w_{5}w_{9}w_{9}w_{9}$
Range Resolution	$w_{7}w_{6}w_{6}w_{6}$		$w_{6}w_{9}w_{9}w_{9}$
	$w_{8}w_{6}w_{6}w_{6}$		$w_{7}w_{7}w_{7}w_{7}$
	$w_{9}w_{6}w_{6}w_{6}$		$w_{8}w_{8}w_{8}w_{8}$
Acquisition or NAT or TM	$w_{6}w_{6}w_{6}w_{6}$		$w_{9}w_{9}w_{9}w_{9}$

**Table 2:** Summary of the performance results of the radar word recognition network.

**Percentage of Missed Pulses**	20%			30%			50%		
**Spur Rate (pulses/s)**	20,000	30,000	40,000	20,000	30,000	40,000	20,000	30,000	40,000
**Recognition Accuracy**	0.997	0.952	0.882	0.998	0.903	0.782	0.930	0.721	0.626

## Data Availability

Not applicable.
